# Preparation of cubic SiC from δ-Na_2_Si_2_O_5_/carbon nanocomposite using cobalt catalyst

**DOI:** 10.1080/14686996.2019.1619479

**Published:** 2019-06-05

**Authors:** Kyeong-Won Park, Oh-Yun Kwon

**Affiliations:** a Department of Chemistry and Faculty of General Education, Gyeongsang National University, Jinju, Republic of Korea; b Department of Chemical and Biomolecular Engineering, Jeonnam National University, Yeosu, Jeonnam, Republic of Korea

**Keywords:** Layered silicate, nanocomposite, silicon carbide, graphite, 10 Engineering and Structural materials, 103 Composites, 301 Chemical syntheses / processing, 503 TEM, STEM, SEM, 104 Carbon and related materials

## Abstract

Silicon carbide (SiC) was prepared by carbothermal reduction of a crystalline-layered sodium silicate (δ-Na_2_Si_2_O_5_)/carbon nanocomposite (LCN), which contained a stacked carbon film embedded with cobalt between the silicate layers. Subsequent sintering of this mixture for 3 h at 1000–1350°C resulted in the formation of graphitic carbon and SiC. Meanwhile, sintering without a cobalt catalyst resulted in the formation of graphitic carbon, regardless of the temperature. The use of a cobalt catalyst allowed the formation of a pure SiC phase at 1350°C. The formed SiC had an irregular worm-like morphology, with a particle size of ~5 µm. The Brunauer-Emmett-Teller surface areas of graphitic carbon and SiC were 28–150 and ~7.0 m^2^/g, respectively. We concluded that graphite and SiC were produced at this low sintering temperature because of the cobalt catalyst, which facilitated nanomixing of carbon and SiO_2_ by sandwiching the carbon films between the silicate layers.

## Graphical abstract

**Figure:**
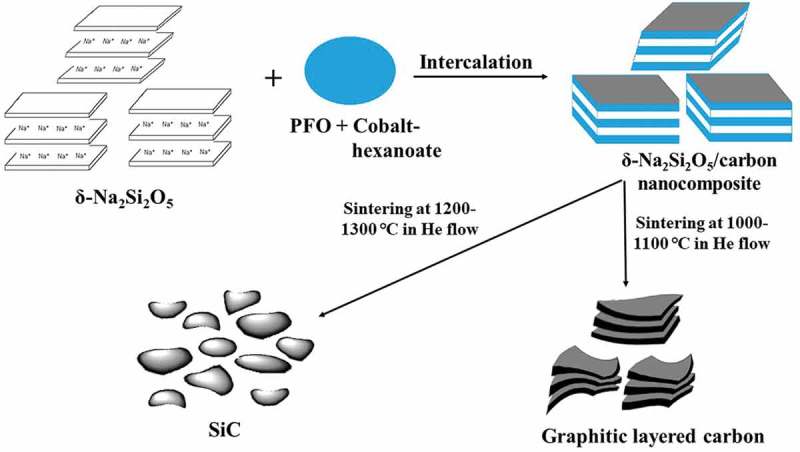

## 1. Introduction

Silicon carbide (SiC) displays many prominent features, such as high hardness, abrasion resistance, melting point, and chemical and thermal shock resistance, as well as possibility to form composites with polymers and low thermal expansion coefficient. Therefore, SiC has been widely used in various industrial applications, such as grinding materials, polishing paste, wear-resistant materials, catalyst supports, filters for molten metals or hot gases, high-temperature structural materials, and reinforcement in composites [1–7].

The synthesis of SiC on an industrial scale takes place through an Acheson process, which involves a carbothermal reduction of sand by petrol coke for several days at about 2400°C [8]. These harsh reaction conditions are required because a heterogeneous mixture is formed between solid sand and solid petrol coke. In order to promote the formation of SiC in a short time at lower temperatures, it would be necessary to create a homogeneous mix with a carbon source on a molecular level. Several reports discuss the effect of the mixing of a carbon source and SiO_2_ on the production of SiC powder [9–19].

Microwave has also been used to prepare SiC because of its advantages of rapid heating, energy saving, environmental protection and lower synthesis temperature [20–23]. However, most of the raw materials that were used to prepare SiC powders by microwave sintering were obtained commercially with little characterization, and the SiC crystal growth mechanism by microwave sintering remains ambiguous.

Recently, SiC nanomaterials with different morphologies such as SiC nanowires [24–34], and mesoporous SiC hollow spheres [35] have been synthesized by several methods [24–27]. Most of the SiC growth methods apply the vapor-liquid-solid (VLS) mechanism, exploiting techniques like the chemical vapor deposition (CVD) and chemical vapor infiltration (CVI) [30–33]. Nanosized SiC-based powders were prepared from selected liquid-phase organosilicon precursors by the aerosol-assisted synthesis, the DC thermal plasma synthesis, and a combination of the two methods [36].

However, most of these studies have not yet been commercially applied due to disadvantages such as high production costs, complex process, and use of expensive raw materials.

In order to overcome this issue, we postulated that intercalation chemistry can be applied as an alternative method for the production of SiC. More specifically, we suggest that layered oxides can form intercalation compounds with some organic and inorganic compounds. The intercalation of these compounds into the interlayer of layered silicate allows the mixing with the silicate framework on a nanoscale, since the layer thickness in layered silicate is 1–2 nm. The resulting expansion of the interfacial surface area causes a chemical reaction and the subsequent formation of an inorganic-organic hybrid. Therefore, the formation of a mix between the carbon source and layered silicate on a nano level would allow the formation of SiC in a short time at a lower temperature. Despite the high applicability of intercalation chemistry for SiC production, it has rarely been discussed in related studies [37,38].

In this study, we report the conversion of a δ-Na_2_Si_2_O_5_/pyrolyzed fuel oil (PFO) nanocomposite to SiC via carbothermal reduction in the presence of cobalt at lower temperature. While various aromatics such as naphthalene, anthracene, pyrene, indene, and indane could be used as carbon precursors in this process, PFO has practical merit as an economical carbon source because it is a by-product of naphtha pyrolysis and contains various polyaromatics (content of more than 60%). In addition, whereas most carbon sources are in the solid phase, PFO is a viscous liquid that could mix homogeneously with a cobalt source such as cobalt 2-hexanoate and with layered silicates. Furthermore, δ-Na_2_Si_2_O_5_ is easily available because it is produced on an industrial scale as a surfactant builder for sequestering of metal ions such as Ca^+^ and Mg^+^. Moreover, since its layers are thinner than those of competitive materials, minerals magadiite, and kenayite, δ-Na_2_Si_2_O_5_ can mix homogeneously with PFO. Overall, the intercalation technique employing a cobalt catalyst presented herein could be very applicable for the commercial production of SiC powder through a simple process using cheap raw materials.

## 2. Experimental details

### 2.1 Materials

Amorphous sodium silicate (molar ratio of SiO_2_/Na_2_O = 2.0) was obtained from Kofran Chem. Co. Cobalt 2-ethylhexanoate ([CH_3_(CH_2_)_3_CH(C_2_H_5_)CO_2_]_2_Co, special grade) was purchased from Sigma-Aldrich, USA. PFO (GS Petroleum Chem. Co., Korea) had an average molecular weight of ~8000 was composed of long-chain hydrocarbons and various polyaromatics (above 60%) with high viscosity (3800–4200 cps).

### 2.2. Sample preparation

#### 2.2.1. δ-na_2_si_2_o_5_

Crystalline-layered sodium silicate (δ-Na_2_Si_2_O_5_) was prepared using amorphous sodium silicate as a starting material through a procedure previously described by J. K. Suh et al. [39] Amorphous sodium silicate was crushed to coarse particles with a jaw crusher and introduced into a vibro-ball mill. The silicate was subsequently ground for 60 min to obtain fine powder. A 50-g sample of the resulting powder was mixed with 11 g of distilled water and left to age in a dryer at 120°C for 60 min to allow for the formation of an aqueous solution of amorphous sodium silicate. The solution was then placed for 30 min in a muffle furnace at 725°C under ambient atmosphere to form crystalline-layered δ-Na_2_Si_2_O_5_. The final product was crushed and used to prepare the nanocomposite with PFO.

#### 2.2.2 Sic

δ-Na_2_Si_2_O_5_ was mixed directly with PFO and cobalt-hexanoate at a weight ratio of δ-Na_2_Si_2_O_5_:PFO:cobalt-hexanoate = 1:1.5:0–1.0. The mixture was left for 24 h at 40°C, allowing the intercalation into the interlayer space. The resulting mixtures of the δ-Na_2_Si_2_O_5_/PFO nanocomposite (LCN) contained 0% and 0.1% cobalt, respectively. The nanocomposites were then loaded onto an alumina boat with a cover and sintered in a programmable quartz tube furnace, where they were kept under 1 L/min He flow for 3 h at the diverse holding temperature range of 1000–1350°C (heating rate: 5°C/min), allowing for the conversion to SiC to take place. The resulting mixture was dispersed for 12 h in an HF (24%) solution to remove the unreacted silicate framework, and then filtered, washed with deionized water, and air-dried to obtain SiC.

### 2.3 Characterization

Powder X-ray diffraction (XRD) measurements were performed using a Rigaku Rotaflex 200B diffractometer equipped with Cu Kα X-ray radiation and a curved crystal graphite monochromator. The scanning electron micrographs (SEMs) were recorded with a JEOL JSM-840A scanning electron microscope. The transmission electron micrographs (TEM) were obtained with a JEOL JEM-200 CX transmission electron microscope operated at 200 kV, using a thin-section technique. The powder samples were embedded in epoxy resin and then sectioned with an ultramicrotome. Nitrogen adsorption/desorption isotherms were determined at 77 K using Micromeritics ASAP 2020. All samples were outgassed at 300°C under vacuum for 4 h. The specific surface areas were determined by the Brunauer-Emmett-Teller (BET) equation.

## 3. Results and discussion

**Figure 1.**
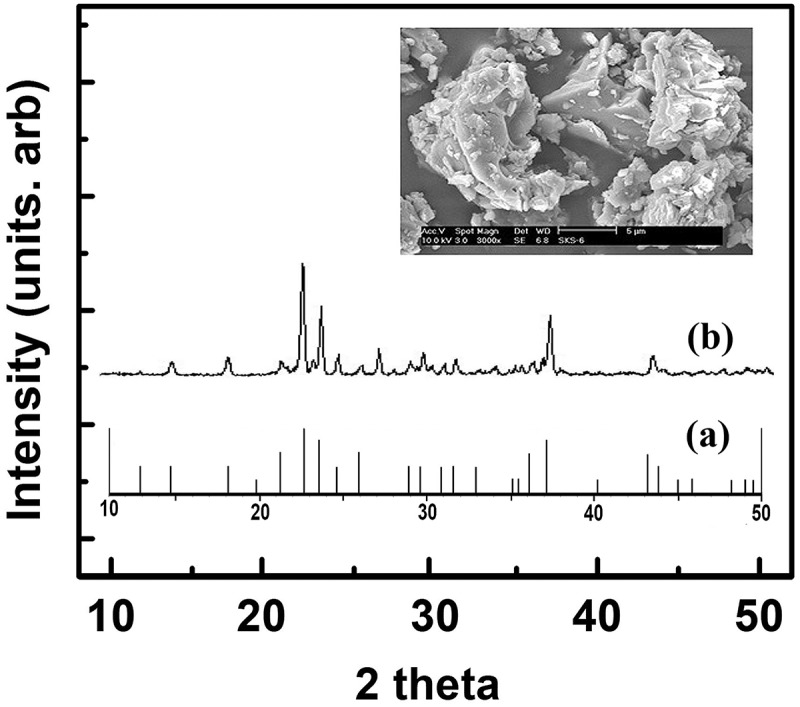
XRD patterns of the (a) reference (JCDPS, No. 29–1233) and (b) δ-Na_2_Si_2_O_5_. Inset: SEM morphology of the synthesized δ-Na_2_Si_2_O_5._

The reaction using amorphous sodium silicate yielded well-crystallized δ-Na_2_Si_2_O_5_. The X-ray powder diffraction pattern of δ-Na_2_Si_2_O_5_ exhibited several reflections (Figure 1), with the peak positions agreeing closely with the reference peaks (JCDPS, No. 29–1233). In addition, the scanning electron micrographs exhibited the typical particle morphology of δ-Na_2_Si_2_O_5_.

In the formation of the intercalation compound, δ-Na_2_Si_2_O_5_ guides PFO to the interlayer spaces, with the Na^+^ ions in the interlayer space acting as electron acceptors for PFO and the π-electrons of the benzene ring in PFO acting as electron donors. The formed intercalation compound was composed of a nanocomposite, in which PFO was sandwiched between silicate layers. Carbonization of the δ-Na_2_Si_2_O_5_/PFO nanocomposite (LCN) produced a δ-Na_2_Si_2_O_5_/carbon nanocomposite with carbon films sandwiched between the silicate layers. Here, the removal of silicate framework from LCN by HF can leave a pure-layered carbon. A successive sintering converted this nanocomposite into SiC via graphite.

Previously, we have reported on the preparation of porous-layered carbon by using Na-kenyaite and PFO [40]. Here, a simple mixing of kenyaite and PFO leads to a kenyaite/PFO intercalation compound, while their pyrolysis results in a kenyaite/carbon nanocomposite with well-developed carbon films located between the silicate layers. Porous-layered carbon with ordered interlayer spaces of about 1 nm could also be obtained by removing the silicate framework using HF.

**Figure 2.**
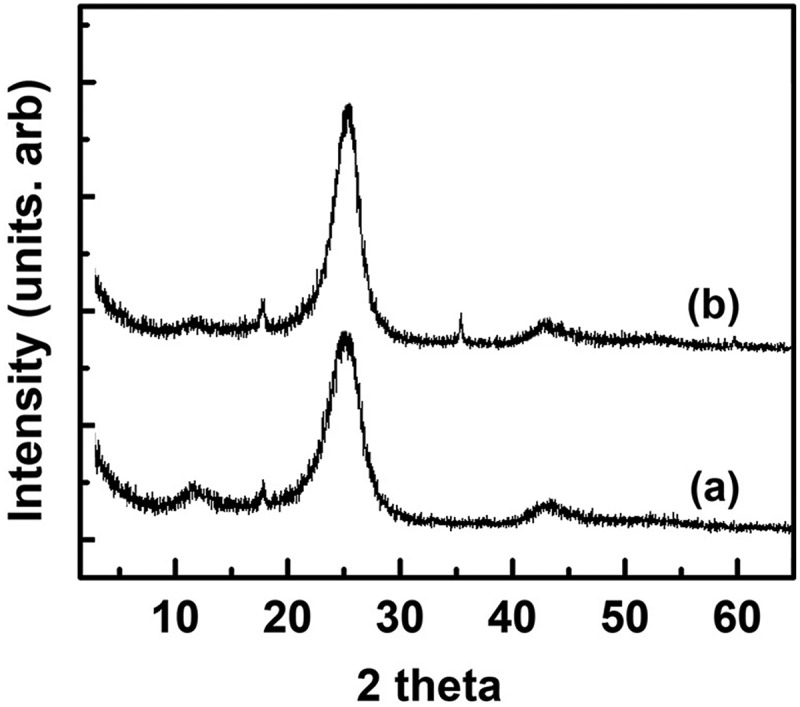
XRD patterns of samples, from which the unreacted silicate framework was removed using HF after sintering LCN without cobalt for 3 h at (a) 1200°C and (b) 1300°C.

Figure 2 shows XRD patterns of the samples, from which the unreacted silicate framework was removed after sintering at 1200–1350°C without cobalt using HF. Characteristic graphite peaks are accompanied by the small broad peak at a diffraction angle (2θ) of 11.5°, which has a basal spacing of about 0.8 nm. Carbonization and sintering of δ-Na_2_Si_2_O_5_/PFO nanocomposite (LCN) result in layered silicate/graphite nanocomposite, which is sandwiched with graphite films between the silicate layers. The removal of silicate framework leaves only sandwiched graphite films with galleries about 1 nm wide. These galleries result in a peak at a diffraction angle (2θ) of 11.5°. The peak is broad and its intensity is low because graphite exists with graphitic carbon comprising amorphous carbon.

**Figure 3.**
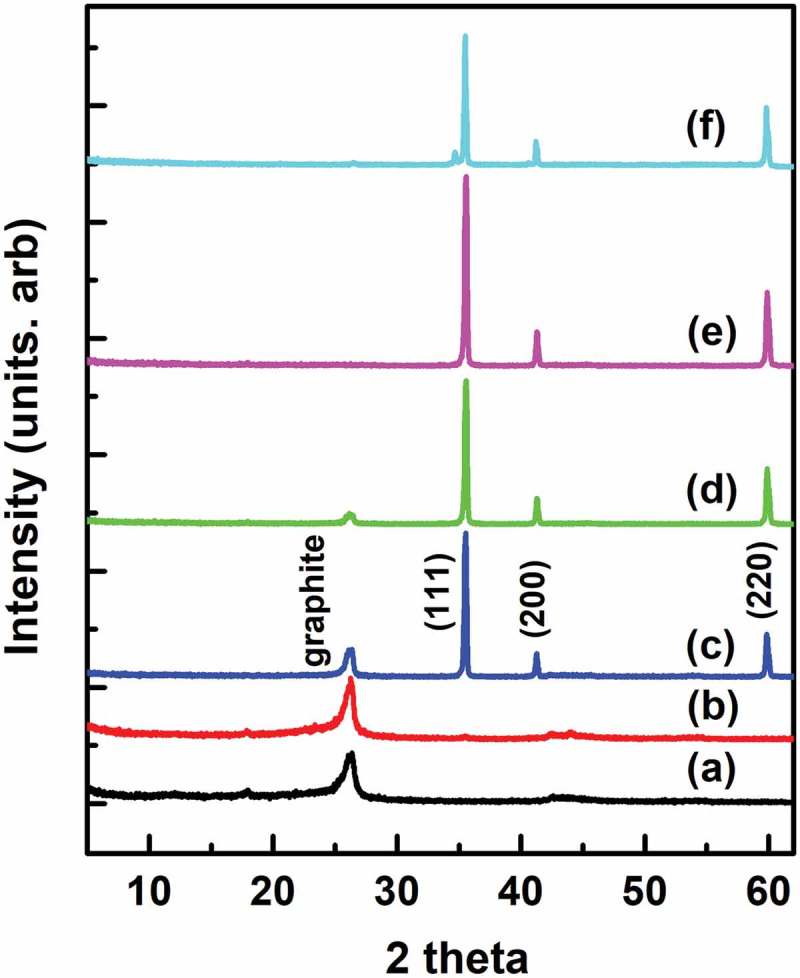
XRD patterns of samples, from which the unreacted silicate framework was removed using HF after sintering LCN with cobalt for 3 h at (a) 1000°C, (b) 1100°C, (c) 1200°C, (d) 1300°C, (e) 1350°C, and (f) pure cubic β-SiC (JCPDS, No. 75–0254).

Figure 3 shows the XRD patterns of samples prepared with 0.1% cobalt, from which the unreacted silicate framework was removed using HF after sintering at 1000–1350°C. As can be seen, graphite peaks appear at 1000–1100°C. In addition, Characteristic SiC peaks appear at diffraction angles of 2θ = 35.66°, 41.41°, 60.01° indexed as the (111), (200), (220) reflections of β-SiC with lattice constant of a = 4.3573 Å (JCPDS, No. 75–0254). The SiC peaks were also accompanied by small graphite peaks (near 2θ = 26°).

Previously, we reported that amorphous carbon in the interlayer space of magadiite and kenyaite could convert to graphite after sintering for 3 h at 900°C in the presence of a cobalt or nickel catalyst [41]. The appearance of SiC peak at 1200°C indicates that the graphite phase formed previously at 1000–1100°C can convert to SiC through a reaction with the silicate framework at temperatures above 1100°C. This indicates that the interlayer space between the silicate layers acted as a nanoreactor for the conversion of amorphous carbon to graphite, with the silicate framework being the silica source in the formation of SiC. Therefore, the effects of the nanomixing and nanoreactor have a great influence on the formation of graphite and the SiC phase in such short sintering times and at low temperatures of 1000–1350°C. Moreover, it confirms that cobalt accelerates the formation of graphite and the SiC phase.

A pure SiC phase with strong XRD peaks was obtained through the reaction for 3 h at 1350°C, elucidating the effect of the sintering temperature on the SiC crystallization. In particular, when compared with the XRD pattern of pure cubic β-SiC (Figure 3(f)), the herein synthesized cubic-SiC (Figure 3(e)) exhibited a well-developed crystallinity.

**Figure 4.**
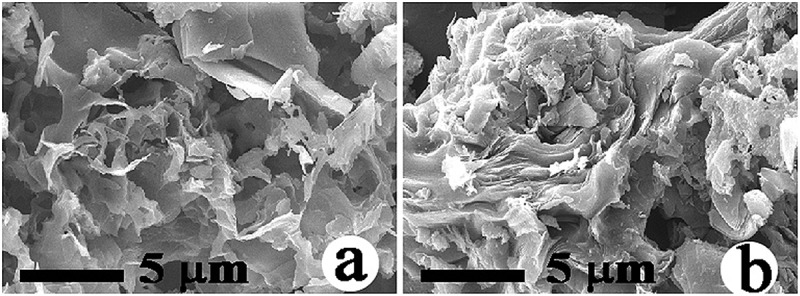
SEM morphologies of the samples, from which the unreacted silicate framework was removed using HF after sintering LCN without cobalt for 3 h at: (a) 1200°C and (b) 1300°C.

Figure 4 exhibits the SEM morphologies of the samples without cobalt, from which the unreacted silicate frame was removed using HF. Notably, the morphology of graphitic carbon was very similar to that (Figure 1) of original δ-Na_2_Si_2_O_5_, indicating that δ-Na_2_Si_2_O_5_ acted as a template during the formation of graphitic carbon.

**Figure 5.**
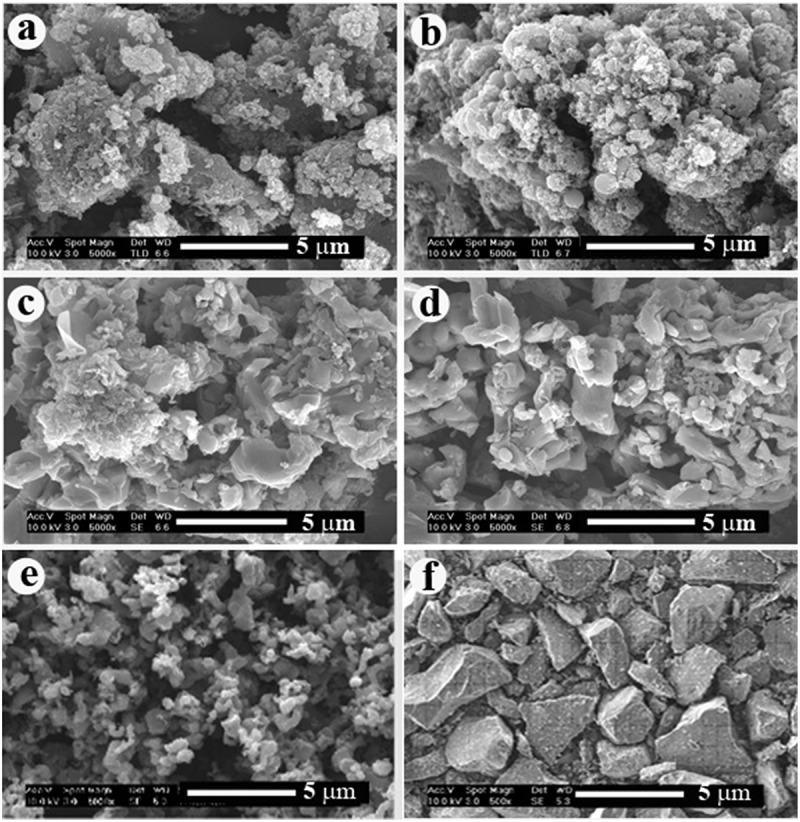
SEM morphologies of samples, from which unreacted silicate framework was removed using HF after sintering LCN with cobalt for 3 h at (a) 1000°C, (b) 1100°C, (c) 1200°C, and (d) 1300°C, (e) 1350°C, and (f) commercial SiC (Sigma-Aldrich).

Figure 5 shows the SEM morphologies of samples with 0.1% cobalt, from which the unreacted silicate framework was removed using HF. The original particle morphology of δ-Na_2_Si_2_O_5_ was broken to bits by the sintering for 3 h at 1000–1100°C, causing the formation of graphitic-layered carbon particles. However, sintering at 1200–1350°C changed the particle morphology to irregular worm-like with a size of ~3 µm due to the formation of SiC. In particular, the size of the as-synthesized SiC particles was smaller than that of the commercial reagent. As mentioned earlier, commercial SiC powder is prepared via the Acheson process, which involves a carbothermal reduction of sand by petrol coke at about 2400°C [8]. This method demands a high temperature and long reaction time, resulting in SiC powder that consists of large grains, which require additional grinding for further use. Therefore, the herein presented method to obtain ultrafine SiC powder would be very industrially appealing.

As shown in Table 1, the BET surface areas of graphitic carbon and SiC samples were in the range of 7.0–150 m^2^/g, depending on the presence of cobalt catalyst and the sintering temperature. The large surface area of graphitic carbon was attributed to the galleries, which were formed from the removal of the silicate templates. Furthermore, increasing the sintering temperature could bring about a large decrease in the surface area, due to the formation of the SiC phase.

**Table 1.** BET surface areas of graphitic carbon and SiC obtained by sintering LCN at various temperatures.

δ-Na_2_Si_2_O_5_/PFO/Cobalt 2-ethylhexanoate (weight ratios)	Sintering time (h)	Sintering temperature (°C)	Surface area (m^2^/g)
1: 1.5: 1.0(0)	3	1000	29
		1100	28
		1200	9 (150)^a^
		1300	7 (90)^a^

**^a^** Numbers in brackets were obtained without using cobalt-hexanoate.

**Figure 6.**
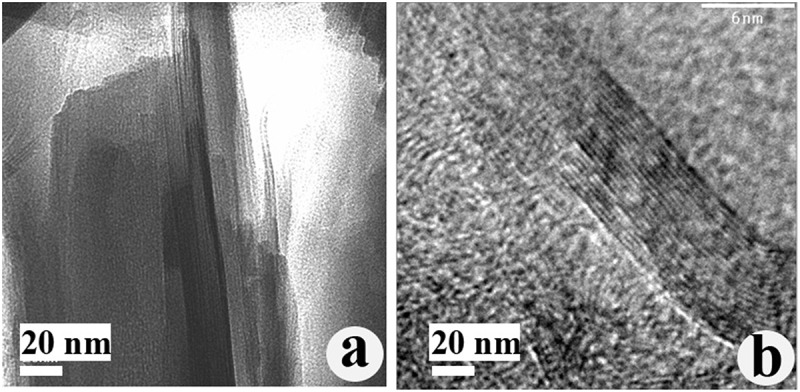
TEM images of (a) layered carbon and (b) graphitic-layered carbon, from which the unreacted silicate framework was removed using HF after sintering LCN with cobalt for 3 h at (a) 600°C and (b) 1100°C.

Figure 6 shows TEM images of layered carbon and graphitic layered carbon. Carbonization of the δ-Na_2_Si_2_O_5_/PFO nanocomposite (LCN) at 600°C produces a δ-Na_2_Si_2_O_5_/carbon nanocomposite. Here, removal of silicate framework left only carbon films sandwiched between the silicate layers (Figure 6(a)). This proves that the carbonization of LCN leads to δ-Na_2_Si_2_O_5_/carbon nanocomposite comprising carbon films sandwiched between the silicate layers. However, the removal of silicate framework after sintering at 1100°C produces graphitic-layered carbon containing two-dimensional galleries (Figure 6(b)). The increase of surface area of graphitic-layered carbon is attributed to the formation of these galleries.

**Figure 7.**
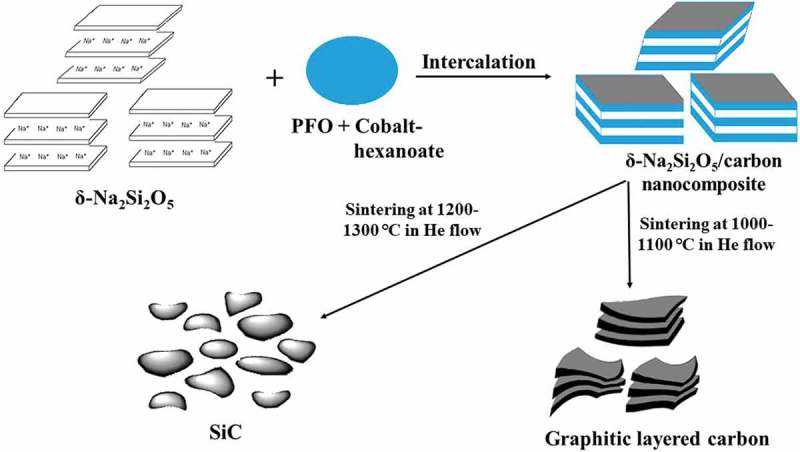
Schematic of the formation of graphitic-layered carbon and SiC.

Figure 7 illustrates the formation of graphitic carbon and the SiC phase. The gallery of δ-Na_2_Si_2_O_5_ was composed of two-dimensional spaces at the nano-scale, which can act as nanoreactors for chemicals introduced into the gallery. This confirms that, in this nanoreactor, PFO could easily be transformed into graphite by sintering at 1000–1100°C, affording a δ-Na_2_Si_2_O_5_/carbon nanocomposite with a carbon film sandwiched between the silicate layers. The removal of the δ-Na_2_Si_2_O_5_ template resulted in graphitic carbon particle with layered structure. Moreover, in the presence of a cobalt catalyst, LCN could also be converted to a well-crystallized SiC phase by sintering above 1350°C. This is attributed to the nanomixing by sandwiching of the δ-Na_2_Si_2_O_5_ framework and carbon source, in which the δ-Na_2_Si_2_O_5_ frame composed of silicate layers acts as a silica source.

## 4. Conclusions

Graphitic carbon and SiC were synthesized through a cobalt-catalyzed carbothermal conversion of LCN in a short sintering time at a temperature range of 1000–1350°C. In the absence of cobalt, the SiC phase did not form even at 1300°C. However, in the presence of cobalt, SiC formed at 1200°C thereby elucidating the effect of cobalt on the formation of the SiC phase. Here, a pure SiC phase was obtained through the reaction for 3 h at 1350°C. The particle morphology of SiC with cobalt was irregular worm-like with a size of ~5 µm. The surface areas of the graphitic-layered carbon and SiC were 28–150 and ~7.0 m^2^/g, respectively.
